# Modification of the Electron Entropy Production in a Plasma

**DOI:** 10.3390/e22090935

**Published:** 2020-08-26

**Authors:** Juan F. García-Camacho, Gonzalo Ares de Parga, Karen Arango-Reyes, Encarnación Salinas-Hernández, Samuel Domínguez-Hernández

**Affiliations:** 1 Escuela Superior de Física y Matemáticas, Instituto Politécnico Nacional, U. P. Adolfo López Mateos, Zacatenco, México City C. P. 07738, Mexico; jgarciac0904@alumno.ipn.mx (J.F.G.-C.); neraky_ar@hotmail.com (K.A.-R.); 2 Escuela Superior de Cómputo, Instituto Politécnico Nacional, Av. Juan de Dios Bátiz esq. Av. Miguel Othón de Mendizábal, Col. Lindavista. Del. Gustavo A. Madero., Ciudad de México C. P. 07738, Mexico; esalinas@ipn.mx; 3 Unidad Profesional Interdisciplinaria en Ingeniería y Tecnologías Avanzadas Instituto Politécnico Nacional, Av. Miguel Othón de Mendizábal s/n. Col. La Escalera. U. P. Adolfo López Mateos, Zacatenco, Del. Gustavo A. Madero, Ciudad de México C. P. 07738, Mexico; sdominguezh@ipn.mx

**Keywords:** plasma, entropy production, Hermite polynomials

## Abstract

A modified expression of the electron entropy production in a plasma is deduced by means of the Kelly equations of state instead of the ideal gas equations of state. From the Debye–Hückel model which considers the interaction between the charges, such equations of state are derived for a plasma and the entropy is deduced. The technique to obtain the modified entropy production is based on usual developments but including the modified equations of state giving the regular result plus some extra terms. We derive an expression of the modified entropy production in terms of the tensorial Hermitian moments $h_{r_{1}\ldots r_{m}}^{\left(m\right)}$ by means of the irreducible tensorial Hermite polynomials.

## 1. Introduction

The entropy production was studied since the middle of the XIX century by Clausius [1]. It has to be highlighted the great exhibition on the subject by Prigogine [2] which permits to understand it from a modern point of view. In the case of a Plasma, a classical review has been done by Hinton and Hazeltine [3]. By assuming a local equilibrium, the rate of change of the entropy can be deduced [4]. The moment method developed by Grad [5] when the distribution function is close to the Boltzmann distribution function, see Equation (20), permits to express such a rate, by using the reducible tensorial Hermite polynomials, in terms of the Hermitian moments ${\tilde{h}}_{r_{1}\ldots r_{m}}^{\left(m\right)}$. Moreover, an improved expression can be derived in terms of the Hermitian moments $h_{r_{1}\ldots r_{m}}^{\left(m\right)}$ by means of the irreducible tensorial Hermite polynomials [4]. In order to highlight the importance of the Hermitian moments, it has to be mentioned that they were included recently by Sonnino et al. [6] to compute the heat loss in L-mode, collisional, tokamak plasmas to test the validity of the sophisticated thermodynamical field theory. However, in the deduction of both the entropy production and, in an implicit form, the Hermitian moments, the equations of state for the Plasma are required but nevertheless, the equations of state of the ideal gas are used as an approximation [4].

On the other hand, by giving different equations of state, it is possible to not abandon the Coulombic interactions of charged plasma particles. The historic paper in Electrochemistry and Plasmas by Debye–Hückel [7] consider such interactions obtaining that the average potential ${\langle \varphi_{\alpha }\left(r\right)\rangle }_{av}$ surrounding a plasma ion bearing a charge $Z_{e}$ is

(1) $${\langle \varphi_{\alpha }\left(r\right)\rangle }_{av}=\left(\frac{Z_{\alpha }e}{r}\right)e^{-\frac{r}{D}},$$

where *D* represents the Debye length

(2) $$D=\sqrt{\frac{kT}{4\pi e^{2}\sum_{\alpha }\frac{N_{\alpha }}{V}Z_{\alpha }^{2}}},$$

where $N_{\alpha }$ represents the number of particles in the volume *V* and the Boltzmann distribution function is considered for obtaining the average. Notice that the same temperature is considered for the ions and the electrons. This will be analyzed in Section 3. In our times, the theory is still being applied to modeling successfully electrolyte solutions [8]. In Plasma Physics, important articles from the middle of last century have analyzed such theory. Kirwood and Poirier [9] have shown that the Coulomb contribution to the virial is

(3) $$-\frac{1}{2}\langle \sum_{i}{\vec{F}}_{i}\cdot {\vec{r}}_{i}\rangle =e^{2}\sum_{\alpha }\frac{N_{\alpha }Z_{\alpha }^{2}V}{4D}=\frac{e^{3}}{2}{\left(\frac{\pi }{kT}\right)}^{\frac{1}{2}}\left(\sum_{\alpha }N_{\alpha }Z_{\alpha }^{2}\right)V.$$

From this, Kelly [10] deduced a new equation of state for the pressure, that is:

(4) $$P=\sum_{\alpha }\frac{N_{\alpha }}{V}kT\left(1-\frac{1}{18N_{D}}\right),$$

where α denotes the different ions and

(5) $$N_{D}=4\pi D^{3}\sum_{\alpha }\frac{N_{\alpha }}{3}.$$

On the other hand, Wergeland [11] relates the corrected term of the pressure $P_{corr}^{e}$ for the electrons with the corrected term of the internal energy as

(6) $$P_{corr}^{e}=\frac{1}{3}\frac{U_{e}}{V}.$$

It should also be noted that over time there have been many attempts to describe equations of state being some of them very sophisticated where the magnetic and electrical fields are included such as the work of Liboff and Lie [12] and recently experimental works for measuring the equations of state [13]. It must be noticed that, considering a small plasma parameter $g={\left(8\pi e^{2}/kT\right)}^{3/2}N/V={\left(D^{3}N/V\right)}^{-1}\ll 1$, by means of the correlation function Krall and Trivelpiece [14] calculated the Gibbs Free Energy and arrived to an expression for the pressure of a plasma similar to the result already obtained by Kelly [10]. Moreover, Krall and Trivelpiece [14] successfully gave a simple expression of the pressure

(7) $$P=2\frac{N}{V}kT\left(1-\frac{g}{48\pi }\right).$$

Furthermore, Wergeland [11] was able to complete the equations of state of a plasma by proposing an expression for the corrected energy (see Equation (6)).

On the other hand, Callen [15] proposed a set of postulates that must be accomplished by the equations of state in order to represent a thermodynamic system (It has to be noticed that in our case the fourth postulate, which represents the third law of thermodynamics, does not have to be satisfied because the analysis of the modified equations of state must be done in a range of temperatures far away from the zero temperature). In this order of ideas, recently, Essex and Andresen [16] exposed an equivalent method to verify that a set of equations of state represents a thermodynamic system. It consists of calculating the eigenvalues of the Hessian derived from the fundamental equation of state, U=U(S,V,N), in the energy picture. One of the eigenvalue must be zero and the others must be positive real numbers. Therefore, we propose the modified equations of state by following Kelly [10] and Wergeland [11]. Then, we verify that they meet the principles of thermodynamics by using the Essex and Andresen method [16] (see [17]). By means of the modified equations of state, the modified electron entropy production can be calculated.

The purpose of this article is threefold:

A—To obtain the modified electron entropy production by using the set of modified equations of state (Section 4) instead of the ideal gas equations of state. Notice that this does not include any effects due to toroidal configurations, rotating plasmas or electrostatic turbulence [18,19]. It has to be noticed that interesting results about bounds for entropy production and H theorems for rf current drive (1D) have been obtained by Bizarro [20]. However, we are not including such kind of analysis in this work.

B—To derive an expression of the modified electron entropy production in terms of the Hermitian moments $h_{r_{1}\ldots r_{m}}^{\left(m\right)}$ by means of the irreducible tensorial Hermite polynomials (Section 4).

C—The modified electron entropy production will contain new terms which are composed by new products of fluxes and forces that may not be so small after an evaluation of them under certain circumstances.

The article is organized as follows: in Section 2, the usual entropy production deduction is presented emphasizing where the ideal gas equations of state are included in order to prepare the modified expression of the entropy production. We also deduce the electron entropy production in terms of the Hermitian moments. In Section 3, the equations of state which include the Coulombic interaction between the electron and the ions, are deduced. To verify that such equations of state describe a thermodynamic system, the entropy is expressed. The thermodynamic properties of the plasma as the heat capacities are deduced. Finally in this section, the eigenvalues of the Hessian are calculated finding a zero eigenvalue which reinforces the idea that we are dealing with a thermodynamic system. In the process, the relaxation coefficients are obtained. In Section 4, we incorporate the modified equations of state to calculate the modified electron entropy production in a Plasma giving the usual results plus extra terms. In addition, by means of the modified equations of state, we derive an expression of the modified electron entropy production in terms of the Hermitian moments $h_{r_{1}\ldots r_{m}}^{\left(m\right)}$ by means of the irreducible tensorial Hermite polynomials. In Section 5, we give some concluding remarks and future work.

## 2. Usual Deduction of the Entropy Production

In order to understand the thermodynamics of a plasma, the entropy represents a fundamental concept and, consequently, it is necessary to have an expression for the entropy production, in particular the entropy production due to the electrons. We present a usual derivation of it where it is emphasized the role played by the equations of the state of the ideal gas. Then, we express the electron entropy production in terms of the Hermitian moments. This is done in order to understand the change that has to be done when the equations of state are different from those of the ideal gas.

### 2.1. Standard Deduction of Entropy Production

Let us begin by considering the equations of state and the average entropy per particle of the ideal gas,

(8) $$P_{\alpha }=n_{\alpha }T_{\alpha },\;U_{\alpha }=\frac{3}{2}NT_{\alpha },\;s_{\alpha }=\ln\left(\frac{T_{\alpha }^{3/2}}{n_{\alpha }}\right),$$

where the subscripts α, $T_{\alpha }$, $U_{\alpha }$, *N*, $n_{\alpha }$ and $s_{\alpha }$ denote the considered species, the temperature, the internal energy, the number of particles, the density of particles and the average entropy per particle of species α where some constants have been withdrawn, respectively. It has to be pointed out that we are putting the Boltzmann constant k=1. The first identity in Equation (8) represents an equation of state which implies that the pressure tensor may be seen as:

(9) $$P_{ij}^{\alpha }=\delta_{ij}P_{\alpha }.$$

Therefore, we can put $s_{\alpha }=s_{\alpha }\left(T_{\alpha },n_{\alpha }\right)$ and consequently

(10) $$\frac{\partial n_{\alpha }s_{\alpha }}{\partial t}=n_{\alpha }\left(\frac{\partial s_{\alpha }}{\partial n_{\alpha }}\frac{\partial n_{\alpha }}{\partial t}+\frac{\partial s_{\alpha }}{\partial T_{\alpha }}\frac{\partial T_{\alpha }}{\partial t}\right)+s_{\alpha }\frac{\partial n_{\alpha }}{\partial t},$$

where no summation Einstein convention has been applied. If we use the Equation (8) in Equation (10), we obtain

(11) $$\frac{\partial n_{\alpha }s_{\alpha }}{\partial t}=n_{\alpha }\left(\frac{\partial s_{\alpha }}{\partial n_{\alpha }}\frac{\partial n_{\alpha }}{\partial t}+\frac{\partial s_{\alpha }}{\partial T_{\alpha }}\frac{\partial (P_{\alpha }/n_{\alpha })}{\partial t}\right)+s_{\alpha }\frac{\partial n_{\alpha }}{\partial t}.$$

Therefore, we arrive at

(12) $$\frac{\partial n_{\alpha }s_{\alpha }}{\partial t}=\left(s_{\alpha }-\frac{5}{2}\right)\frac{\partial n_{\alpha }}{\partial t}+\frac{1}{T_{\alpha }}\frac{\partial }{\partial t}\left(\frac{3}{2}P_{\alpha }\right).$$

From the Fokker–Planck Equation [3], which includes the Fokker–Planck collision operator, it can be derived that

(13) $$\frac{\partial }{\partial t}\left(\frac{3}{2}p_{\alpha }+\frac{\left(m_{\alpha }n_{\alpha }u_{\alpha }^{2}\right)}{2}\right)+\nabla \cdot Q_{\alpha }=Q_{\alpha }+u_{\alpha }\cdot \left(F_{\alpha }+e_{\alpha }n_{\alpha }E\right),$$

where $u_{\alpha }$ and E represent the averaged velocity of the species α and the electric field, respectively; the friction force $F_{\alpha }$ is

(14) $$F_{\alpha }=\int m_{\alpha }vC_{\alpha }d^{3}v,$$

with $C_{\alpha }$ the collision term of the species α; the collisional rate of heat exchange $Q_{\alpha }$ is

(15) $$Q_{\alpha }=\int {\left(v-u_{\alpha }\right)}^{2}\frac{m_{\alpha }}{2}C_{\alpha }d^{3}v,$$

(notice that $C_{\alpha }$ does not depend on the equations of state) and the energy flux $Q_{\alpha }$ is

(16) $$Q_{\alpha }=\int \frac{m_{\alpha }v^{2}}{2}vf_{\alpha }d^{3}v,$$

where $f_{\alpha }$ represents the distribution function. By defining the heat flux as

(17) $$q_{\alpha }=\int \frac{m_{\alpha }}{2}{\left(v-u_{\alpha }\right)}^{2}\left(v-u_{\alpha }\right)f_{\alpha }d^{3}v,$$

We know that

(18) $$\int \frac{m_{\alpha }v^{2}}{2}f_{\alpha }dv=\int \frac{m_{\alpha }{\left(v-u_{\alpha }\right)}^{2}}{2}f_{\alpha }dv+\frac{m_{\alpha }n_{\alpha }u_{\alpha }}{2}=3p_{\alpha }/2+\frac{m_{\alpha }n_{\alpha }u_{\alpha }^{2}}{2}.$$

The thermal velocity $v_{Te}^{2}$ is related with the temperature by [3]

(19) $$\frac{v_{Te}^{2}}{2}=\frac{T_{e}}{m_{e}},$$

and the following approximation for the distribution function,

(20) $$f_{\alpha }=f_{\alpha o}\left(1+\chi_{\alpha }\right),\;with\;\chi_{\alpha }≃\frac{u_{\alpha }}{v_{T\alpha }}\ll 1,$$

where $f_{\alpha 0}$ represents the Boltzmann function or any distribution function which must decrease sufficiently fast at infinity and permits to obtain

(21) $$\frac{m_{\alpha }n_{\alpha }u_{\alpha }^{2}}{2}=n_{\alpha }T_{\alpha }{\left(\frac{u_{\alpha }}{v_{T\alpha }}\right)}^{2}\Rightarrow \frac{m_{\alpha }n_{\alpha }u_{\alpha }}{2}≃0.$$

Consequently,

(22) $$\int \frac{m_{\alpha }v^{2}}{2}f_{\alpha }dv=3p_{\alpha }/2.$$

Taking into account Equation (20), we have

(23) $$\int v_{r}^{2}f_{\alpha }dv\approx \int v_{k}^{2}f_{\alpha o}d^{3}v=\frac{p_{\alpha }}{m}$$

by using Equation (8) in Equation (17), we obtain (neglecting $u_{\alpha }^{2}$)

(24) $$q_{\alpha }=Q_{\alpha }-\frac{5P_{\alpha }u_{\alpha }}{2}.$$

By using the approximation described in Equation (20), which implies that the term $\partial (m_{\alpha }n_{\alpha }u_{\alpha }^{2})/\partial t$ can be neglected, and by using Equation (13), Equation (12) can be expressed as

(25) $$\frac{\partial n_{\alpha }s_{\alpha }}{\partial t}=\left(s_{\alpha }-5/2\right)\frac{\partial n_{\alpha }}{\partial t}+\frac{\left(Q_{\alpha }+u_{\alpha }\cdot \left(F_{\alpha }+e_{\alpha }n_{\alpha }E\right)\right)}{T_{\alpha }}-\frac{1}{T_{\alpha }}\nabla \cdot Q_{\alpha }.$$

On the other hand, by using Equation (8) in Equation (24), we have

(26) $$q_{\alpha }=Q_{\alpha }-\frac{5}{2}n_{\alpha }T_{\alpha }u_{\alpha }.$$

Substituting Equation (26) into Equation (25), we obtain

(27) $$\begin{array}{cc} \frac{\partial n_{\alpha }s_{\alpha }}{\partial t}= & \left(s_{\alpha }-5/2\right)\frac{\partial n_{\alpha }}{\partial t}+\frac{Q_{\alpha }+u_{\alpha }\cdot \left(F_{\alpha }+e_{\alpha }n_{\alpha }E\right)}{T_{\alpha }}\\ & -\frac{1}{T_{\alpha }}\nabla \cdot q_{\alpha }-\frac{5}{2}\nabla \cdot \left(n_{\alpha }u_{\alpha }\right)-\frac{5}{2T_{\alpha }}n_{\alpha }u_{\alpha }\cdot \nabla T_{\alpha }. \end{array}$$

Considering the equation of continuity

(28) $$\frac{\partial n_{\alpha }}{\partial t}+\nabla \cdot \left(n_{\alpha }u_{\alpha }\right)=0,$$

we arrive at

(29) $$\frac{\partial n_{\alpha }s_{\alpha }}{\partial t}=-s_{\alpha }\nabla \cdot \left(n_{\alpha }u_{\alpha }\right)-\frac{1}{T_{\alpha }}\nabla \cdot q_{\alpha }+\frac{Q_{\alpha }+u_{\alpha }\cdot \left(F_{\alpha }+e_{\alpha }n_{\alpha }E\right)}{T_{\alpha }}-\frac{5}{2T_{\alpha }}n_{\alpha }u_{\alpha }\cdot \nabla T_{\alpha }.$$

Considering the equation of state, Equation (8), we obtain

(30) $$\frac{\partial n_{\alpha }s_{\alpha }}{\partial t}=-s_{\alpha }\nabla \cdot \left(n_{\alpha }u_{\alpha }\right)-\frac{1}{T_{\alpha }}\nabla \cdot q_{\alpha }+\frac{Q_{\alpha }+u_{\alpha }\cdot \left(F_{\alpha }+e_{\alpha }n_{\alpha }E\right)}{T_{\alpha }}-\frac{5}{2T_{\alpha }}n_{\alpha }u_{\alpha }\cdot \nabla \left(\frac{p_{\alpha }}{n_{\alpha }}\right).$$

That is:

(31) $$\begin{array}{cc} \frac{\partial n_{\alpha }s_{\alpha }}{\partial t}= & -s_{\alpha }\nabla \cdot \left(n_{\alpha }u_{\alpha }\right)-\frac{1}{T_{\alpha }}\nabla \cdot q_{\alpha }+\frac{Q_{\alpha }+u_{\alpha }\cdot \left(F_{\alpha }+e_{\alpha }n_{\alpha }E\right)}{T_{\alpha }}\\ & -\frac{5}{2T_{\alpha }}u_{\alpha }\cdot \nabla \left(p_{\alpha }\right)+\frac{5}{2}u_{\alpha }\cdot \nabla n_{\alpha }. \end{array}$$

We obtain

(32) $$\frac{\partial n_{\alpha }s_{\alpha }}{\partial t}+\nabla \cdot \left(s_{\alpha }n_{\alpha }u_{\alpha }+\frac{q_{\alpha }}{T_{\alpha }}\right)=-\frac{q_{\alpha }}{T_{\alpha }^{2}}\cdot \nabla T_{\alpha }+\frac{Q_{\alpha }+u_{\alpha }\cdot \left(F_{\alpha }+e_{\alpha }n_{\alpha }E\right)}{T_{\alpha }}-\frac{u_{\alpha }}{T_{\alpha }}\cdot \nabla p_{\alpha }.$$

We define the flux vector

(33) $$J_{\alpha s}=s_{\alpha }n_{\alpha }u_{\alpha }+\frac{q_{\alpha }}{T_{\alpha }},$$

and considering that

(34) $$\sigma_{\alpha }=\partial /\partial t\left(n_{\alpha }s_{\alpha }\right)+\nabla \cdot J_{\alpha s},$$

we arrive at:

(35) $$\sigma_{\alpha }=-\frac{q_{\alpha }}{T_{\alpha }^{2}}\cdot \nabla T_{\alpha }+\frac{Q_{\alpha }+u_{\alpha }\cdot \left(F_{\alpha }+e_{\alpha }n_{\alpha }E\right)}{T_{\alpha }}-\frac{u_{\alpha }}{T_{\alpha }}\cdot \nabla p_{\alpha }.$$

This result coincides with the one obtained by Hinton and Hazeltine [3].

### 2.2. Electron Entropy Production in Terms of the Hermitian Moments

Let us give the Balescus [4] expression of the electron entropy production:

(36) $$\sigma^{e}=\frac{n_{e}}{\tau_{e}}\left(h_{r}^{\left(1\right)}g_{r}^{\left(1\right)}+h_{r}^{e\left(3\right)}g_{r}^{e\left(3\right)}+h_{rs}^{e\left(2\right)}g_{rs}^{e\left(2\right)}\right),$$

where some dimensionless Hermitian moments and dimensionless source terms are defined as:

(37) $$\begin{array}{ccc} h_{r}^{\left(1\right)} & = & {\left(\frac{m_{e}}{T_{e}}\right)}^{\frac{1}{2}}\frac{j_{r}}{en_{e}},\;h_{r}^{e\left(3\right)}=\sqrt{\frac{2}{5}}{\left(\frac{m_{e}}{T_{e}}\right)}^{\frac{3}{2}}\frac{q_{r}^{e}}{m_{e}n_{e}},\;h_{rs}^{e\left(2\right)}=\frac{\pi_{rs}}{\sqrt{2}n_{e}T_{e}},\\ g_{r}^{\left(1\right)} & = & \tau_{e}{\left(\frac{m_{e}}{T_{e}}\right)}^{\frac{1}{2}}\left(\frac{eE_{r}}{m_{e}}-w_{e}\varepsilon_{rmn}u_{m}b_{n}+\frac{\nabla_{r}P_{\alpha }}{m_{e}n_{e}}\right),\\ g_{r}^{e\left(3\right)} & = & -\sqrt{\frac{5}{2}}\tau_{e}{\left(\frac{T_{e}}{m_{e}}\right)}^{\frac{1}{2}}\frac{\nabla_{r}T_{e}}{T_{e}},\;g_{rs}^{e\left(2\right)}=-\sqrt{2}\tau_{e}\nabla_{r}u_{s}, \end{array}$$

with the relaxation electron time $\tau_{e}$,

(38) $$\tau_{e}=\frac{3}{4\sqrt{2\pi }}\frac{m_{e}^{\frac{1}{2}}T_{e}^{\frac{3}{2}}}{z^{2}e^{4}n_{e}\ln\Lambda }\;with\;\ln\Lambda =\ln\left(\frac{\frac{3}{2}\left(T_{e}+T_{i}\right)D}{Ze^{2}}\right),$$

being lnΛ the Coulomb logarithm; and

(39) $$w_{e}=-\frac{eB}{m_{e}c},\;and\;B=Bb.$$

Let us constrain the Plasma to two species: the electrons and one type of ion. $\pi_{rs}$ are defined below in Equation (41), j in Equation (43) and u in Equation (46). First, some approximations made by Balescu should be set; in order to obtain Equation (36), the collisonal rate of heat exchange and the friction force must be considered as negligible, that is:

(40) $$Q_{e}≃0\;and\;F_{e}≃0.$$

If we want to compare Equation (36) with Equation (35), it is necessary to consider the fundamental approximation done obtaining Equation (35) which is (see Equation (9)), that is:

(41) $$P_{ij}^{e}=P_{e}\delta_{ij}+\pi_{ij}\;with\;\pi_{ij}≃0.$$

Applying this last equation, Equation (36) turns to be

(42) $$\sigma_{e}=\frac{j\cdot E}{T_{e}}-w\frac{j\cdot \left(u\times b\right)}{T_{e}m_{e}e}+\frac{j\cdot \nabla P_{e}}{en_{e}}-\frac{q\cdot \nabla T_{e}}{T_{e}^{2}}.$$

Therefore, from the one fluid model, we have

(43) $$u_{i}-u_{e}=\frac{j}{en_{e}},$$

where we have considered neutral total charge. Since

(44) $$u_{i}\ll u_{e},$$

we have

(45) $$-u_{e}=\frac{j}{en_{e}}.$$

On the other hand, the average velocity is defined as

(46) $$u=\frac{m_{e}n_{e}u_{e}+m_{i}n_{i}u_{i}}{m_{e}n_{e}+m_{i}n_{i}}≃u_{i},$$

because $(m_{e}/m_{i})≃0$. Therefore, by substituting Equations (45) and (46) in Equation (42), for the electron entropy production, we obtain

(47) $$\sigma_{e}=-\frac{en_{e}u_{e}\cdot E}{T_{e}}+w_{e}\frac{n_{e}u_{e}\cdot \left(u_{i}\times b\right)}{T_{e}m_{e}}-\frac{u_{e}}{T_{e}}\cdot \nabla P_{e}-\frac{q_{e}}{T_{e}^{2}}\cdot \nabla T_{e}.$$

Since $u_{e}$ collinear with $u_{i}$, we arrive at

(48) $$\sigma_{e}=-\frac{en_{e}u_{e}\cdot E}{T_{e}}-\frac{u_{e}}{T_{e}}\cdot \nabla P_{e}-\frac{q_{e}}{T_{e}^{2}}\cdot \nabla T_{e}.$$

Making a comparison between Equations (35), (36) and (48), we can see that they coincide if we consider that Equations (40) and (41) are satisfied. This method is used to obtain the modified electron entropy production when we modify the equations of state in Section 4.

## 3. Equations of State for the Kelly Plasma

For more than 80 years, there has been much interest in Plasma Physics due to its applications in Tokamaks and Astrophysics. However, under non-relativistic conditions, the equations of state for an ideal gas are used as the first approximation to calculate the equations of balance, the moment equations and thermodynamic flows [3,4]. Nevertheless, among other proposals, Kelly [10] gave an equation of state for a Plasma with different species relating the pressure *P*, the number of particles for each species $N_{i}$, the Volume *V* and the temperature *T*. However, one equation of state is not enough to describe a system composed by many species without given other equations of state. This point was partially corrected by Wergeland [11] by including an expression for the corrected internal energy due to Debye–Hückel theory [7] where the energy is obtained by making an average of the Coulomb energy.

Our purpose in this section consists of completing the set of equations of state for a plasma with two species: electrons and one type of ion. Since in many situations in Plasma Physics, it is just necessary to consider only the electrons, we analyze the Kelly equation for one species. We will obtain the electron entropy for a plasma composed of electrons where the effect of the ions are considered just in the equations of state. We obtain the entropy as a function of the volume *V*, the number of electrons *N* and the temperature *T*. However, it is not possible to write explicitly the entropy as a function of the internal energy *U*, the volume *V* and the number of electrons *N* (or the internal energy as a function of the entropy *S*, the volume *V* and the number of electrons *N*). Accordingly, by using a technique based on Maxwell’s relations [17], we obtain the eigenvalues of the Hessian in order to check the viability of the system. Moreover, The nonzero eigenvalues are related with the relaxation times which are very important in Plasma Physics. With these results, we can affirm that the new set of equations of state describes a thermodynamic system and can be used instead of the ideal gas equations of state in the calculation of the electron entropy production.

### 3.1. Preliminaries

In the case of considering only electrons and ions in a quasi-neutral plasma, the Kelly equation for the partial pressure of the electrons can be written as

(49) $$P_{e}=\frac{N}{V}kT\left(1-\frac{1}{18N_{D}}\right)=\frac{N}{V}kT\left(1-\frac{1}{24\frac{N}{V}{\left(\frac{VkT}{4\pi Ne^{2}}\right)}^{\frac{3}{2}}}\right),$$

where

(50) $$N_{D}=\frac{4}{3}\pi D^{3}\frac{N}{V}\;D={\left(\frac{k}{4\pi e^{2}Z_{1}^{2}}\right)}^{\frac{1}{2}}\frac{V^{\frac{1}{2}}T^{\frac{1}{2}}}{N^{\frac{1}{2}}}={\left(\frac{kTV}{4\pi Ne^{2}}\right)}^{1/2}.$$

It is necessary to note that *D* is the Debye’s length and $N_{D}$ represents the number of particles contained in a Debye’s sphere. Following Wergeland [11] and Equation (6), the equations of state are:

(51) $$P=\frac{NkT}{V}-\frac{N^{\frac{3}{2}}}{3V^{\frac{3}{2}}T^{\frac{1}{2}}}\left(\frac{\pi^{\frac{1}{2}}e^{3}}{k^{\frac{1}{2}}}\right),$$

and

(52) $$U=\frac{3}{2}NkT-\frac{N^{\frac{3}{2}}}{V^{\frac{1}{2}}T^{\frac{1}{2}}}\left(\frac{\pi^{\frac{1}{2}}e^{3}}{k^{\frac{1}{2}}}\right).$$

It is interesting that we can obtain the heat capacity $C_{V,N}$, that is:

(53) $$C_{V,N}={\left[\frac{\partial U}{\partial T}\right]}_{V,N}=\frac{3}{2}Nk+\frac{N^{\frac{3}{2}}}{2V^{\frac{1}{2}}T^{\frac{3}{2}}}\left(\frac{\pi^{\frac{1}{2}}e^{3}}{k^{\frac{1}{2}}}\right).$$

It has to be highlighted that if we consider the expression of the usual entropy production, Equation (48), and under certain circumstances it is considered to cancel, the modified entropy production, Equation (94), may not vanish. Although the term may be considered in first instance as negligible, because the correction is of the order of $N_{D}^{-1}$ which for plasma is very small, we will see that due to the new terms, the modified entropy production may not be so small.

### 3.2. Entropy

Due to the similarity with the relationship between energy and pressure in the case of photons, we can propose a correction for the entropy given by the following expression

(54) $$S_{corr}=\frac{U_{corr}}{3T}.$$

Notice that due to the expression of the corrected energy in our case (see Equation (52)), there is a factor 1/3 and not a factor 4/3 as it happens in the photon case. Then, we can propose the total entropy as

(55) $$S=S_{ig}+S_{corr},$$

where $S_{ig}$ represents the entropy for a null charge, e=0, that is the ideal gas. Therefore,

(56) $$\begin{array}{cc} S= & S_{ig}+\frac{U_{corr}}{3T}\\ = & S_{ig}-\frac{N^{\frac{3}{2}}}{3V^{\frac{1}{2}}T^{\frac{3}{2}}}\left(\frac{\pi^{\frac{1}{2}}e^{3}}{k^{\frac{1}{2}}}\right). \end{array}$$

If we calculate the Helmholtz free energy $A=A_{ig}+U_{corr}-TS_{corr}$ and deduce the pressure by means of $P=-{\left[\frac{\partial A}{\partial V}\right]}_{T,N}$, we obtain the equations of state described in Equation (51) which partially verifies that we are dealing with a thermodynamic system. However, this is not enough to assure that the set of equations of state, Equations (51) and (52), represents a thermodynamic system. Indeed, it is necessary to show that entropy as a fundamental equation is a first degree homogeneous function and that the temperature is positive, or simply obtain the eigenvalues of the Hessian showing one of them is null and the others are positive real quantities.

### 3.3. the Hessian of the Kelly Plasma

Since the electron entropy and the internal energy cannot be written explicitly as fundamental equations (S=S(U,V,N) or U=U(S,V,N)), we proceed to calculate the eigenvalues of the Hessian. Since the Hessian is constituted by entrees of this kind ${\left[\partial^{2}U/\partial X_{i}\partial X_{j}\right]}_{X_{k}}$, where the $X_{i}$ represents the extensive variables of the system, we can use thermodynamic relations as the Maxwell’s relations in order to obtain the Hessian. For example, if we have an expression of T=T(S,V,N), it will be sufficient in order to calculate

(57) $${\left[\frac{\partial^{2}U}{\partial S\partial S}\right]}_{V,N}={\left[\frac{\partial T}{\partial S}\right]}_{V,N}.$$

Then, the eingenvalues are obtained by following this technique [17] and they are the following: we found an eigenvalue λ=0, and the other two eigenvalues are

(58) $$\lambda_{\pm }=\alpha \pm \beta ,$$

The eigenvalues α and β have been calculated by Arango-Reyes and Ares de Parga [17] and are positive real quantities for the range of temperatures of the Plasma and they are related with the relaxation times. Therefore, the system described by Equations (51), (52) and (56) represents a thermodynamic system and it can be used to calculate the electron entropy production.

## 4. Modified Electron Entropy Production

Once we have a new set of equations of state for the electrons in a Plasma, Equations (51), (52) and (56), we can calculate the new expression of the electron entropy production applying the same procedure than in the usual case but using the new set of equations of state. In our treatment, we rewrite the equations of state for the electrons as (in this section we return to consider k = 1 as in Section 2):

(59) $$P_{e}=n_{e}T_{e}-\frac{\gamma n_{e}^{3/2}}{T_{e}^{1/2}},$$

where

(60) $$\gamma =\frac{\pi^{1/2}e^{3}}{3},$$

and Equation (41) has been considered. In addition,

(61) $$s=\ln\left(\frac{T_{e}^{3/2}}{n_{e}}\right)-\frac{\gamma n_{e}^{1/2}}{T_{e}^{3/2}}.$$

### 4.1. Deduction of the Modified Entropy Production

Let us begin by calculating as in Section 2,

(62) $$\frac{\partial P_{e}}{\partial t}=n_{e}\left(1+\frac{1}{2}\gamma n_{e}^{1/2}T_{e}^{-3/2}\right)\frac{\partial T_{e}}{\partial t}+T_{e}\left(1-\frac{3}{2}\gamma n_{e}^{1/2}T_{e}^{-3/2}\right)\frac{\partial n_{e}}{\partial t}.$$

Therefore, we can express $\frac{\partial T_{e}}{\partial t}$ as

(63) $$\frac{\partial T_{e}}{\partial t}=\frac{2}{3}\frac{1}{n_{e}\left(1+\frac{1}{2}\gamma n_{e}^{1/2}T_{e}^{-3/2}\right)}\frac{\partial \left(\frac{3}{2}P_{e}\right)}{\partial t}-\frac{T_{e}\left(1-\frac{3}{2}\gamma n_{e}^{1/2}T_{e}^{-3/2}\right)}{n_{e}\left(1+\frac{1}{2}\gamma n_{e}^{1/2}T_{e}^{-3/2}\right)}\frac{\partial n_{e}}{\partial t}$$

With these results, we can proceed to calculate $\frac{\partial s_{e}}{\partial n_{e}}$ and $\frac{\partial s_{e}}{\partial T_{e}}$ with the help of Equation (61), we have

(64) $$\frac{\partial s_{e}}{\partial n_{e}}=-\frac{1}{n_{e}}\left(1+\frac{\gamma n_{e}^{1/2}}{2}T_{e}^{-3/2}\right),$$

and

(65) $$\frac{\partial s_{e}}{\partial T_{e}}=\frac{3}{2T_{e}}\left(1+\gamma n_{e}^{1/2}T_{e}^{-3/2}\right)$$

By using Equation (24) and using the new equations of state, we arrive at

(66) $$Q_{e}=q_{e}+\frac{5}{2}n_{e}T_{e}u_{e}-\frac{5}{2}\frac{\gamma n_{e}^{3/2}}{T_{e}^{1/2}}u_{e}.$$

Once we have these identities, we can develop $\frac{\partial n_{e}s_{e}}{\partial t}$; that is, substituting Equations (63)–(65) into Equation (10), we have

(67) $$\frac{\partial n_{e}s_{e}}{\partial t}=n_{e}\left(\begin{array}{c} \left(s_{e}-\frac{1}{n_{e}}\left(1+\frac{\gamma }{2n_{e}^{1/2}}T_{e}^{-3/2}\right)-\frac{3}{2}\frac{1}{T_{e}}\left(1+\gamma n_{e}^{1/2}T_{e}^{-3/2}\right)\frac{T_{e}\left(1-\frac{3}{2}\gamma n_{e}^{1/2}T_{e}^{-3/2}\right)}{n_{e}\left(1+\frac{1}{2}\gamma n_{e}^{1/2}T_{e}^{-3/2}\right)}\right)\frac{\partial n_{e}}{\partial t}\\ +\frac{1}{T_{e}}\left(1+\gamma n_{e}^{1/2}T_{e}^{-3/2}\right)\frac{1}{n_{e}\left(1+\frac{1}{2}\gamma n_{e}^{1/2}T_{e}^{-3/2}\right)}\frac{\partial \left(\frac{3}{2}P_{e}\right)}{\partial t} \end{array}\right).$$

By using Equation (13), considering too that the term proportional to $u_{e}^{2}$ is neglected, we have

(68) $$\frac{\partial n_{e}s_{e}}{\partial t}=n_{e}\left(\begin{array}{c} \left(s_{e}-\frac{1}{n_{e}}\left(1+\frac{\gamma }{2n_{e}^{1/2}}T_{e}^{-3/2}\right)-\frac{3}{2}\frac{1}{T_{e}}\left(1+\gamma n_{e}^{1/2}T_{e}^{-3/2}\right)\frac{T_{e}\left(1-\frac{3}{2}\gamma n_{e}^{1/2}T_{e}^{-3/2}\right)}{n_{e}\left(1+\frac{1}{2}\gamma n_{e}^{1/2}T_{e}^{-3/2}\right)}\right)\frac{\partial n_{e}}{\partial t}\\ +\frac{\left(1+\gamma n_{e}^{1/2}T_{e}^{-3/2}\right)}{n_{e}T_{e}\left(1+\frac{1}{2}\gamma n_{e}^{1/2}T_{e}^{-3/2}\right)}\left(Q_{e}+u_{e}\cdot \left(F_{e}-en_{e}E\right)-\nabla \cdot Q_{e}\right) \end{array}\right)$$

Substituting Equation (66) in the last equation, we obtain:

(69) $$\frac{\partial n_{e}s_{e}}{\partial t}=n_{e}\left(\begin{array}{c} \left(s_{e}-\frac{1}{n_{e}}\left(1+\frac{\gamma }{2n_{e}^{1/2}}T_{e}^{-3/2}\right)-\frac{3}{2}\frac{1}{T_{e}}\left(1+\gamma n_{e}^{1/2}T_{e}^{-3/2}\right)\frac{T_{e}\left(1-\frac{3}{2}\gamma n_{e}^{1/2}T_{e}^{-3/2}\right)}{n_{e}\left(1+\frac{1}{2}\gamma n_{e}^{1/2}T_{e}^{-3/2}\right)}\right)\frac{\partial n_{e}}{\partial t}\\ +\frac{\left(1+\gamma n_{e}^{1/2}T_{e}^{-3/2}\right)}{n_{e}T_{e}\left(1+\frac{1}{2}\gamma n_{e}^{1/2}T_{e}^{-3/2}\right)}\left(Q_{e}+u_{e}\cdot \left(F_{e}-en_{e}E\right)-\nabla \cdot \left(q_{e}+\frac{5}{2}n_{e}T_{e}u_{e}-\frac{5}{2}\frac{\gamma n_{e}^{3/2}}{T_{e}^{1/2}}u_{e}\right)\right) \end{array}\right)$$

Let us calculate first $\nabla \cdot \left(n_{e}T_{e}u_{e}\right)$ and $\nabla \cdot \left(\frac{\gamma n_{e}^{3/2}}{T_{e}^{1/2}}u_{e}\right)$:

(70) $$\nabla \cdot \left(n_{e}T_{e}u_{e}\right)=n_{e}u_{e}\cdot \nabla \cdot T_{e}+T_{e}\nabla \cdot n_{e}u_{e},$$

and

(71) $$\begin{array}{cc} \nabla \cdot \left(\frac{\gamma n_{e}^{3/2}}{T_{e}^{1/2}}u_{e}\right)= & n_{e}u_{e}\cdot \left(\frac{1}{2}n_{e}^{-1/2}T_{e}^{-1/2}\nabla n_{e}-\frac{1}{2}n_{e}^{1/2}T_{e}^{-3/2}\nabla T_{e}\right)\\ & +\frac{n_{e}^{1/2}}{T_{e}^{1/2}}\nabla \cdot \left(n_{e}u_{e}\right). \end{array}$$

Then, let us calculate $\nabla P_{e}$,

(72) $$\nabla P_{e}=n_{e}\left(1+\frac{1}{2}\gamma n_{e}^{1/2}T_{e}^{-3/2}\right)\nabla T_{e}+T_{e}\left(1-\frac{3}{2}\gamma n_{e}^{1/2}T_{e}^{-3/2}\right)\nabla n_{e}.$$

We can write explicitly $\nabla T_{e}$,

(73) $$\nabla T_{e}=\frac{1}{n_{e}\left(1+\frac{1}{2}\gamma n_{e}^{1/2}T_{e}^{-3/2}\right)}\nabla P_{e}-\frac{T_{e}\left(1-\frac{3}{2}\gamma n_{e}^{1/2}T_{e}^{-3/2}\right)}{n_{e}\left(1+\frac{1}{2}\gamma n_{e}^{1/2}T_{e}^{-3/2}\right)}\nabla n_{e}.$$

By using Equations (70) and (72), we can calculate

(74) $$\begin{array}{cc} -\nabla \cdot \left(\frac{5}{2}n_{e}T_{e}u_{e}-\frac{5}{2}\frac{\gamma n_{e}^{3/2}}{T_{e}^{1/2}}u_{e}\right)= & -\frac{5}{2}\left(1+\frac{1}{2}\gamma n_{e}^{1/2}T_{e}^{-3/2}\right)n_{e}u_{e}\cdot \nabla T_{e}\\ & -\frac{5}{2}\left(1-\gamma n_{e}^{1/2}T_{e}^{-3/2}\right)\nabla \cdot \left(n_{e}u_{e}\right)\\ & +\frac{5}{4}\gamma n_{e}^{1/2}T_{e}^{-1/2}n_{e}u_{e}\cdot \nabla n_{e} \end{array}$$

By substituting Equation (73) into Equation (74), we obtain

(75) $$\begin{array}{cc} -\nabla \cdot \left(\frac{5}{2}n_{e}T_{e}u_{e}-\frac{5}{2}\frac{\gamma n_{e}^{3/2}}{T_{e}^{1/2}}u_{e}\right)= & \frac{5}{2}T_{e}\left(1-\gamma n_{e}^{1/2}T_{e}^{-3/2}\right)\left(u_{e}\cdot \nabla n_{e}-\nabla \cdot \left(n_{e}u_{e}\right)\right)\\ & -\frac{5}{2}u_{e}\cdot \nabla P_{e} \end{array}$$

By using this last equation in Equation (69), we arrive at

(76) $$\frac{\partial n_{e}s_{e}}{\partial t}=\left(\begin{array}{c} \left(s_{e}-\left(1+\frac{\gamma }{2n_{e}^{1/2}}T_{e}^{-3/2}\right)-\frac{3}{2}\left(1+\gamma n_{e}^{1/2}T_{e}^{-3/2}\right)\frac{\left(1-\frac{3}{2}\gamma n_{e}^{1/2}T_{e}^{-3/2}\right)}{1+\frac{1}{2}\gamma n_{e}^{1/2}T_{e}^{-3/2}}\right)\frac{\partial n_{e}}{\partial t}\\ +\frac{\left(1+\gamma n_{e}^{1/2}T_{e}^{-3/2}\right)}{n_{e}\left(1+\frac{1}{2}\gamma n_{e}^{1/2}T_{e}^{-3/2}\right)}\left(\begin{array}{c} \frac{Q_{e}+u_{e}\cdot \left(F_{e}-en_{e}E\right)}{T_{e}}\\ \frac{5}{2}\left(1-\gamma n_{e}^{1/2}T_{e}^{-3/2}\right)\left(u_{e}\cdot \nabla n_{e}-\nabla \cdot \left(n_{e}u_{e}\right)\right)\\ -\frac{5}{2T_{e}}u_{e}\cdot \nabla P_{e} \end{array}\right) \end{array}\right).$$

Let us now make an approximation

(77) $$\gamma n_{e}^{1/2}T_{e}^{-3/2}\ll 1,$$

which is a natural approximation in a Tokamak [21], such that

(78) $$\frac{\left(1+\gamma n^{1/2}T^{-3/2}\right)}{\left(1+\frac{1}{2}\gamma n^{1/2}T^{-3/2}\right)}\approx 1+\frac{1}{2}\gamma n^{1/2}T^{-3/2},$$

Therefore,

(79) $$\frac{\partial n_{e}s_{e}}{\partial t}=\left(\begin{array}{c} \left(s_{e}-\left(1+\frac{\gamma }{2n_{e}^{1/2}}T_{e}^{-3/2}\right)-\frac{3}{2}\left(1+\frac{1}{2}\gamma n_{e}^{1/2}T_{e}^{-3/2}\right)\frac{\left(1-\frac{3}{2}\gamma n_{e}^{1/2}T_{e}^{-3/2}\right)}{\left(1+\frac{1}{2}\gamma n_{e}^{1/2}T_{e}^{-3/2}\right)}\right)\frac{\partial n_{e}}{\partial t}\\ +\left(1+\frac{1}{2}\gamma n_{e}^{1/2}T_{e}^{-3/2}\right)\left(\begin{array}{c} \frac{Q_{e}+u_{e}\cdot \left(F_{e}-en_{e}E\right)}{T}\\ +\frac{5}{2}\left(1-\gamma n_{e}^{1/2}T_{e}^{-3/2}\right)\left(u_{e}\cdot \nabla n_{e}-\nabla \cdot \left(n_{e}u_{e}\right)\right)\\ -\frac{5}{2T_{e}}u_{e}\cdot \nabla P_{e} \end{array}\right) \end{array}\right)$$

Simplifying, we arrive at

(80) $$\frac{\partial n_{e}s_{e}}{\partial t}=\left(\begin{array}{c} \left(s_{e}-\frac{5}{2}+\frac{\gamma }{n_{e}^{1/2}}T_{e}^{-3/2}\right)\frac{\partial n_{e}}{\partial t}+\left(1+\frac{1}{2}\gamma n_{e}^{1/2}T_{e}^{-3/2}\right)\left(\frac{Q_{e}+u_{e}\cdot \left(F_{e}-en_{e}E\right)-\nabla \cdot q_{e}}{T_{e}}\right)\\ -\left(1+\frac{1}{2}\gamma n_{e}^{1/2}T_{e}^{-3/2}\right)\frac{5}{2}u_{e}\cdot \nabla P_{e}+\frac{5}{2}\left(1-\frac{1}{2}\gamma n_{e}^{1/2}T_{e}^{-3/2}\right)\left(u_{e}\cdot \nabla n_{e}-\nabla \cdot \left(n_{e}u_{e}\right)\right) \end{array}\right)$$

If we use the equation of continuity in Equation (28), we obtain

(81) $$\begin{array}{cc} \frac{\partial n_{e}s_{e}}{\partial t}= & -s_{e}\nabla \cdot \left(n_{e}u_{e}\right)+\frac{Q_{e}+u_{e}\cdot \left(F_{e}-en_{e}E\right)}{T_{e}}\\ & -\frac{\nabla \cdot q_{e}}{T_{e}}-\frac{5}{2T_{e}}u_{e}\cdot \nabla P_{e}+\frac{5}{2}u_{e}\cdot \nabla n_{e}\\ & +\frac{1}{2}\gamma n_{e}^{1/2}T_{e}^{-3/2}\left(\begin{array}{c} \frac{1}{2}\nabla \cdot \left(n_{e}u_{e}\right)+\frac{Q_{e}+u_{e}\cdot \left(F_{e}-en_{e}E\right)}{T_{e}}\\ -\frac{\nabla \cdot q_{e}}{T_{e}}-\frac{5}{2T_{e}}u_{e}\cdot \nabla P_{e}-\frac{5}{2}u_{e}\cdot \nabla n_{e} \end{array}\right). \end{array}$$

On the other hand, from Equation (59), we can obtain

(82) $$\nabla n_{e}=\frac{1}{\left(T_{e}-\frac{3}{2}\frac{\gamma n_{e}^{1/2}}{T_{e}^{1/2}}\right)}\nabla P_{e}-\frac{\left(n_{e}+\frac{1}{2}\frac{\gamma n_{e}^{1/2}}{T_{e}^{3/2}}\right)}{\left(T_{e}-\frac{3}{2}\frac{\gamma n_{e}^{1/2}}{T_{e}^{1/2}}\right)}\nabla T_{e}.$$

If in Equation (82), we use the approximation of Equation (77), we have

(83) $$\nabla n_{e}=\frac{1}{T_{e}}\left(T_{e}+\frac{3}{2}\gamma n_{e}^{1/2}T_{e}^{-3/2}\right)\nabla P_{e}-\frac{n_{e}}{T_{e}}\left(1+2\gamma n_{e}^{1/2}T_{e}^{-3/2}\right)\nabla T_{e}.$$

By making the scalar product of the last equation with $\left(5/2\right)\left(T_{e}-\frac{1}{2}\gamma n_{e}^{1/2}T_{e}^{-3/2}\right)u_{e}$, we arrive at

(84) $$\begin{array}{cc} \frac{5}{2}\left(T_{e}-\frac{1}{2}\gamma n_{e}^{1/2}T_{e}^{-3/2}\right)u_{e}\cdot \nabla n_{e}= & \frac{5}{2}\left(T_{e}+\gamma n_{e}^{1/2}T_{e}^{-3/2}\right)\frac{1}{T_{e}}u_{e}\cdot \nabla P_{e}\\ & -\frac{5}{2}\left(1+\frac{3}{2}\gamma n_{e}^{1/2}T_{e}^{-3/2}\right)\frac{n_{e}}{T_{e}}u_{e}\cdot \nabla T_{e}. \end{array}$$

By using Equation (84) in Equation (81), we obtain

(85) $$\begin{array}{cc} \frac{\partial n_{e}s_{e}}{\partial t}= & \left(\left(s_{e}-\frac{1}{4}\gamma n_{e}^{1/2}T_{e}^{-3/2}\right)\nabla \cdot \left(n_{e}u_{e}\right)\right)\\ & +\left(1+\frac{1}{2}\gamma n_{e}^{1/2}T_{e}^{-3/2}\right)\left(\frac{Q_{e}+u_{e}\cdot \left(F_{e}-en_{e}E\right)-\nabla \cdot q_{e}}{T_{e}}\right)\\ & +\frac{5}{4}\left(\gamma n_{e}^{1/2}T_{e}^{-3/2}\right)\frac{u_{e}}{T_{e}}\cdot \nabla P_{e}-\frac{5}{2}\left(1+\frac{3}{2}\gamma n_{e}^{1/2}T_{e}^{-3/2}\right)\frac{n_{e}}{T_{e}}u_{e}\cdot \nabla T_{e}. \end{array}$$

In order to put our attention in the equation of balance, we must consider the following identity:

(86) $$\begin{array}{cc} -s_{e}\nabla \cdot n_{e}u_{e}= & -\nabla \cdot \left(s_{e}n_{e}u_{e}\right)-\left(1+2\gamma n_{e}^{1/2}T_{e}^{-3/2}\right)\frac{1}{T_{e}}u_{e}\cdot \nabla P_{e}\\ & +\left(\frac{5}{2}+4\gamma n_{e}^{1/2}T_{e}^{-3/2}\right)\frac{n_{e}}{T_{e}}u_{e}\cdot \nabla T_{e} \end{array}$$

If we substitute Equation (86) into Equation (85), we have

(87) $$\begin{array}{cc} \frac{\partial n_{e}s_{e}}{\partial t}+\nabla \cdot \left(s_{e}n_{e}u_{e}+\frac{q_{e}}{T_{e}}\right)= & \frac{1}{T_{e}}\left(u_{e}\cdot \nabla P_{e}\right)\left(1+\frac{3}{4}\gamma n_{e}^{1/2}T_{e}^{-3/2}\right)\\ & -\left(\frac{q_{e}}{T_{e}}\cdot \nabla T_{e}\right)\left(1+\frac{1}{2}\gamma n_{e}^{1/2}T_{e}^{-3/2}\right)\\ & +\left(1+\frac{1}{2}\gamma n_{e}^{1/2}T_{e}^{-3/2}\right)\left(\frac{Q_{e}+u_{e}\cdot \left(F_{e}-en_{e}E\right)}{T_{e}}\right)\\ & +\frac{1}{4}\gamma n_{e}^{1/2}T_{e}^{-3/2}\frac{n_{e}}{T_{e}}u_{e}\cdot \nabla T_{e}+\frac{1}{4}\gamma n_{e}^{1/2}T_{e}^{-3/2}\nabla \cdot \left(n_{e}u_{e}\right)\\ & -\frac{1}{2}\gamma n_{e}^{1/2}T_{e}^{-3/2}\nabla \cdot \left(\frac{q_{e}}{T_{e}}\right). \end{array}$$

Simplifying, we arrive at

(88) $$\begin{array}{cc} & \frac{\partial n_{e}s_{e}}{\partial t}+\nabla \cdot \left(n_{e}u_{e}\left(s_{e}-\frac{1}{4}\gamma n_{e}^{1/2}T_{e}^{-3/2}\right)+\frac{q_{e}}{T_{e}}\left(1+\frac{1}{2}\gamma n_{e}^{1/2}T_{e}^{-3/2}\right)\right)\\ = & -\frac{u_{e}}{T_{e}}\cdot \left(\nabla P_{e}\right)\left(1+\frac{7}{8}\gamma n_{e}^{1/2}T_{e}^{-3/2}\right)-\frac{q_{e}}{T_{e}^{2}}\cdot \left(\nabla T_{e}\right)\left(1+\frac{3}{2}\gamma n_{e}^{1/2}T_{e}^{-3/2}\right)\\ & +\left(1+\frac{1}{2}\gamma n_{e}^{1/2}T_{e}^{-3/2}\right)\left(\frac{Q_{e}+u_{e}\cdot \left(F_{e}-en_{e}E\right)}{T_{e}}\right)\\ & +\frac{1}{4}\gamma n_{e}^{1/2}T_{e}^{-3/2}\frac{q_{e}}{n_{e}T_{e}^{2}}\cdot \nabla P_{e}+\frac{3}{4}\gamma n_{e}^{1/2}T_{e}^{-3/2}\frac{n_{e}}{T_{e}}u_{e}\cdot \nabla T_{e}. \end{array}$$

We can define now

(89) $$J_{esm}=n_{e}u_{e}\left(s_{e}-\frac{1}{4}\gamma n_{e}^{1/2}T_{e}^{-3/2}\right)+\frac{q_{e}}{T_{e}}\left(1+\frac{1}{2}\gamma n_{e}^{1/2}T_{e}^{-3/2}\right).$$

Since

(90) $$\sigma_{em}=\frac{\partial }{\partial t}\left(n_{e}s_{e}\right)+\nabla \cdot J_{esm},$$

we have

(91) $$\begin{array}{cc} \sigma_{em}= & -\frac{u_{e}}{T_{e}}\cdot \left(\nabla P_{e}\right)\left(1+\frac{7}{8}\gamma n_{e}^{1/2}T_{e}^{-3/2}\right)-\frac{q_{e}}{T_{e}^{2}}\cdot \left(\nabla T_{e}\right)\left(1+\frac{3}{2}\gamma n_{e}^{1/2}T_{e}^{-3/2}\right)\\ & +\left(1+\frac{1}{2}\gamma n_{e}^{1/2}T_{e}^{-3/2}\right)\left(\frac{Q_{e}+u_{e}\cdot \left(F_{e}-en_{e}E\right)}{T_{e}}\right)\\ & +\frac{1}{4}\gamma n_{e}^{1/2}T_{e}^{-3/2}\frac{q_{e}}{n_{e}T_{e}^{2}}\cdot \nabla P_{e}+\frac{3}{4}\gamma n_{e}^{1/2}T_{e}^{-3/2}\frac{n_{e}}{T_{e}}u_{e}\cdot \nabla T_{e}. \end{array}$$

Notice that by using Equations (33) and (35), we arrive at

(92) $$J_{esm}=J_{es}+\frac{1}{2}\gamma n_{e}^{1/2}T_{e}^{-3/2}\left(\frac{q_{e}}{T_{e}}-\frac{n_{e}u_{e}}{2}\right)$$

and

(93) $$\sigma_{em}=\sigma_{e}+\frac{1}{2}\gamma n_{e}^{1/2}T_{e}^{-3/2}\left(\begin{array}{c} -3\frac{q_{e}}{T_{e}^{2}}\cdot \left(\nabla T_{e}\right)+\left(\frac{Q_{e}+u_{e}\cdot \left(F_{e}-en_{e}E\right)}{T_{e}}\right)\\ -\frac{7}{4}\frac{u_{e}}{T_{e}}\cdot \left(\nabla P_{e}\right)+\frac{1}{2}\frac{q_{e}}{n_{e}T_{e}^{2}}\cdot \nabla P_{e}+\frac{3}{2}\frac{n_{e}}{T_{e}}u_{e}\cdot \nabla T_{e} \end{array}\right).$$

### 4.2. Deduction of the Modified Electron Entropy Production in Terms of the Hermitian Moments

Let us consider the modified entropy production, Equations (91) or (93) but with the approximations described in Equations (40), (41) and (77), that is:

(94) $$\sigma_{em}=\sigma_{e}+\frac{1}{2}\gamma n_{e}^{1/2}T_{e}^{-3/2}\left(\begin{array}{c} -3\frac{q_{e}}{T_{e}^{2}}\cdot \left(\nabla T_{e}\right)-en_{e}\frac{u_{e}\cdot E}{T_{e}}\\ -\frac{7}{4}\frac{u_{e}}{T_{e}}\cdot \left(\nabla P_{e}\right)+\frac{1}{2}\frac{q_{e}}{n_{e}T_{e}^{2}}\cdot \nabla P_{e}+\frac{3}{2}\frac{n_{e}}{T_{e}}u_{e}\cdot \nabla T_{e} \end{array}\right)$$

If we compare the usual entropy production $\sigma_{e}$, Equation (48), and the modified entropy production $\sigma_{em}$, Equation (94), we can see that in the correction term there are the same three products of flows by forces except for the factor $\left(\frac{1}{2}\gamma n_{e}^{1/2}T_{e}^{-3/2}\right)$ and their respective different coefficients as −3 and −7/4 unless for two new terms $\frac{1}{2}\frac{q_{e}}{n_{e}T_{e}^{2}}\cdot \nabla P_{e}$ and $\frac{3}{2}\frac{n_{e}}{T_{e}}u_{e}\cdot \nabla T_{e}$. Under certain circumstances, the usual energy production may vanish as for example: $q_{e}=0,E=0$ and $\nabla P_{e}=0$, which implies that $\sigma_{e}=0$. In such a case due to the resemblance of three of the terms in the correction term of the entropy production, the modified entropy production, for typical conditions of a Tokamak [21], can be approximated to

(95) $$\sigma_{em}≃\left(\frac{1}{2}\gamma n_{e}^{1/2}T_{e}^{-3/2}\right)\frac{3}{2}\frac{n_{e}}{T_{e}}u_{e}\cdot \nabla T_{e}≃4\times 10^{-10}6\frac{10^{14}}{10^{-8}}≃24\times 10^{12}u_{e}\cdot \nabla T_{e}.$$

Therefore, even if $u_{e}\cdot \nabla T_{e}$ is very small, $\sigma_{em}$ cannot be neglected and consequently the corrections done to the equations of state lead us to results that in certain circumstances are not negligible.

Furthermore, these last expressions show that the change in the expression of entropy production can be significant when equations of state very different from the ideal gas equations are used. Moreover, new products of fluxes and forces, $\left(\frac{1}{2}\gamma n_{e}^{1/2}T_{e}^{-3/2}\right)\frac{1}{2}\frac{q_{e}}{n_{e}T_{e}^{2}}\cdot \nabla P_{e}$ and $\left(\frac{1}{2}\gamma n_{e}^{1/2}T_{e}^{-3/2}\right)\frac{3}{2}\frac{n_{e}}{T_{e}}u_{e}\cdot \nabla T_{e}$, appear in the modified entropy production which represent an important result if we want to analyze the system from the point of view of the Thermodynamics of Irreversible Processes [2,22].

By using Equations (37) and (45) and reminding that $u^{e}$ is collinear with $u^{i}$, we have

(96) $$\begin{array}{ccc} u_{r}^{e} & = & -{\left(\frac{T_{e}}{m_{e}}\right)}^{\frac{1}{2}}h_{r}^{\left(1\right)},\;q_{r}^{e}=m_{e}n_{e}\sqrt{\frac{5}{2}}{\left(\frac{T_{e}}{m_{e}}\right)}^{\frac{3}{2}}h_{r}^{e\left(3\right)},\\ g_{r}^{\left(1\right)} & = & \tau_{e}{\left(\frac{m_{e}}{T_{e}}\right)}^{\frac{1}{2}}\left(\frac{eE_{r}}{m_{e}}+\frac{1}{m_{e}n_{e}}\frac{\partial P_{e}}{\partial x_{r}}\right),\\ g_{r}^{e\left(3\right)} & = & -\sqrt{\frac{5}{2}}\tau_{e}{\left(\frac{T_{e}}{m_{e}}\right)}^{\frac{1}{2}}\frac{1}{T_{e}}\frac{\partial T_{e}}{\partial x_{r}}. \end{array}$$

Therefore, in this case, we can express the regular electron entropy production $\sigma_{e}$ as

(97) $$\sigma_{e}=\frac{n_{e}}{\tau_{e}}\left(h_{r}^{\left(1\right)}g_{r}^{\left(1\right)}+h_{r}^{e\left(3\right)}g_{r}^{e\left(3\right)}\right).$$

For the modified electron entropy production we need to redefine the $g\overset{´}{}s$ terms, that is

(98) $$g_{mr}^{\left(1\right)}=g_{r}^{\left(1\right)}-\tau_{e}{\left(\frac{m_{e}}{T_{e}}\right)}^{\frac{1}{2}}\frac{1}{2}\gamma n_{e}^{1/2}T_{e}^{-3/2}\left(\frac{eE_{r}}{m_{e}}-\frac{7}{4}\frac{1}{m_{e}n_{e}}\frac{\partial P_{e}}{\partial x_{r}}+\frac{3}{2}\frac{1}{m_{e}}\frac{\partial T_{e}}{\partial x_{r}}\right),$$

and

(99) $$g_{mr}^{e\left(3\right)}=g_{r}^{e\left(3\right)}+\tau_{e}\sqrt{\frac{5}{2}}{\left(\frac{m_{e}}{T_{e}}\right)}^{\frac{1}{2}}\frac{1}{2}\gamma n_{e}^{1/2}T_{e}^{-3/2}\left(3\frac{1}{T_{e}}\frac{\partial T_{e}}{\partial x_{r}}+\frac{1}{2}\frac{1}{n_{e}T_{e}}\frac{\partial P_{e}}{\partial x_{r}}\right).$$

We arrive at

(100) $$\sigma_{em}=\frac{n_{e}}{\tau_{e}}\left(h_{r}^{\left(1\right)}g_{mr}^{\left(1\right)}+h_{r}^{e\left(3\right)}g_{mr}^{e\left(3\right)}\right).$$

## 5. Concluding Remarks

We obtain an expression for the modified electron entropy production by means of the Kelly equations of state within the approximation described by Equations (40) and (41) but keeping the constraint in Equation (77). It has to be noticed that within our approximations the ion entropy production does not suffer any modification.

The new terms in the modified entropy production represent new products of fluxes and forces. Such products may not be so negligible in some circumstances which implies that some analysis can be done with respect the Linear Irreversible Thermodynamics [22]. The modifications done to the expression of the energy production in terms of dimensionless Hermitian moments and dimensionless source terms, Equation (100), may suggest that when distribution functions are different from the Boltzmann distribution function, the irreducible tensorial Hermite polynomials will not be useful as Izacard [23] proposed by making a generalization of the Hermite polynomial representation.

The next step consists of obtaining the modified electron entropy production without using such approximations. Such expression will include the moment $h_{r}^{e\left(2\right)}$ by considering that the collisonal rate of heat exchange $Q_{e}$, the friction force $F_{e}$ and the traceless pressure tensor $\pi_{ij}^{e}$ do not have to be neglected.
